# Educational differences in diabetes and diabetes self-management behaviours in WHO SAGE countries

**DOI:** 10.1186/s12889-021-12131-7

**Published:** 2021-11-17

**Authors:** Karen E. Lamb, David Crawford, Lukar E. Thornton, Sheikh M. Shariful Islam, Ralph Maddison, Kylie Ball

**Affiliations:** 1 Deakin University, Institute for Physical Activity and Nutrition (IPAN), School of Exercise and Nutrition Sciences, Geelong, Australia; 2 Murdoch Children’s Research Institute, Royal Children’s Hospital, Parkville, VIC Australia; 3 Melbourne School of Population and Global Health, The University of Melbourne, Parkville, VIC Australia

**Keywords:** Diabetes mellitus, Global Health, Developing countries, Cohort, Adult

## Abstract

**Background:**

Diabetes mellitus represents a substantial global health challenge, with prevalence rising in low- and middle-income countries (LMICs). Although diabetes is known to follow a socioeconomic gradient, patterns in LMICs are unclear. This study examined associations between education and diabetes, and diabetes self-management behaviours, in six LMICs.

**Methods:**

Cross-sectional data for 31,780 participants from China, Ghana, India, Mexico, Russia, and South Africa from the World Health Organization Study on Global AGEing and adult health (SAGE) study were used. Participants aged ≥50 years completed face-to-face interviews between 2007 and 2010. Participants self-reported diabetes diagnosis, physical activity, sedentary time, fruit and vegetable consumption, any special diet/program for diabetes, whether they were taking insulin for diabetes and number of years of education. Height, weight, waist, and hip circumference were measured. Country-specific survey-weighted log-binomial regression models were fitted to examine associations between the number of years of education and self-reported diabetes diagnosis (primary analysis). In secondary analyses, among those with a self-reported diabetes diagnosis, generalised linear regression models were fitted to examine associations between education and i) physical activity, ii) sedentary time, iii) fruit and vegetable consumption, iv) special diet for diabetes, v) taking insulin, vi) BMI, vii) waist circumference and viii) hip circumference.

**Results:**

There was strong evidence of an association between years of education and diabetes diagnosis in Ghana (RR = 1.09, 95% CI: 1.06–1.13) and India (RR = 1.09, 95% CI: 1.07–1.12) only. In India, greater years of education was associated with higher leisure physical activity, fruit and vegetable intake, rates following a special diet or taking insulin, but also higher mean BMI, waist and hip circumference. Relationships between education and self-management behaviours were rarely seen in the other countries.

**Conclusions:**

Associations between education and diabetes, and behavioural self-management (India only) was more evident in the two least developed (Ghana and India) of the WHO SAGE countries, indicating increasing diabetes diagnosis with greater numbers of years of education. The lack of gradients elsewhere may reflect shifting risk from higher to lower educated populations. While there was some suggestion that self-management behaviours were greater with increased education in India, this was not observed in the other countries.

**Supplementary Information:**

The online version contains supplementary material available at 10.1186/s12889-021-12131-7.

## Background

Diabetes mellitus represents a substantial global health challenge, with prevalence in the world’s adult population doubling from 4.7 to 8.5% between 1980 and 2014 [1]. Research has shown a considerable proportion of the world’s population with diabetes live in low-and middle-income countries (LMICs), with the highest proportion of adults under 50 years with diabetes found in low-income countries (67%) [2], where prevalence has risen most rapidly since 1980 [1]. The prevalence of diabetes is estimated to increase by 69% in LMICs and 20% in high-income countries (HICs) between 2010 and 2030 [3]. The global cost of diabetes was estimated at $1.31 trillion in 2015, with the economic burden as a percentage of Gross Domestic Product (GDP) larger in LMICs than in HICs [4].

In high-income countries, diabetes follows a socioeconomic gradient, with the most socioeconomically disadvantaged at greatest risk [5]. Socioeconomic gradients among LMICs are less clear, and of interest given that these countries shoulder a growing healthcare burden associated with diabetes [1]. Evidence suggest that socioeconomic gradients in non-communicable diseases (NCDs) might be different in LMICs compared to HICs [6, 7]. Further, NCDs risk factors often change over time, with countries following an epidemiological transition associated with increasing development [8]. The transition of countries towards a higher level of development may be associated with a reversal of the socioeconomic gradient in diabetes risk. For example, at a national level, Xu et al. [9] found that diabetes prevalence increased with increasing country-level socioeconomic status in developing countries, but decreased with increasing status in developed countries. When considering individual-level socioeconomic position, evidence shows, for example, an attenuation between 2002 and 2008 of the high concentration of diabetes cases amongst the most advantaged in South Africa [10]. However, evidence remains mixed in other LMICs [11, 12]. In one of few studies which examined socioeconomic gradients conducted in multiple countries, Tyrovolas et al. (2015) [13] found that education was more strongly associated with diabetes in some countries (e.g., Ghana, India, Poland) than others (Mexico, Russia), and suggested that level of development may moderate education-diabetes associations. However, their study was focussed on examining associations between obesity, diabetes and disability and thus their findings on socioeconomic gradients were exploratory and did not account for potential confounders of the association. Furthermore, that study also excluded any focus on behavioural self-management. Contrasting findings across LMICs is hampered by challenges obtaining comparable estimates across studies due to varying definitions of diabetes; data sources and representativeness; years of data collection; and confounders considered [14].

Effective self-management of diabetes requires compliance with medication regimens, as well as modifying behavioural risk factors including maintaining a healthy weight, being physically active, and eating a healthy diet. Little is known about the extent to which people living with diabetes in LMICs engage in behaviours to help manage their condition, nor about socioeconomic gradients in these behaviours. If gradients follow patterns observed among HICs, this could contribute to worsening disease progression amongst those who are more socioeconomically disadvantaged if this group has poorer engagement in diabetes self-care, as shown elsewhere [15, 16].

Examining socioeconomic gradients in diabetes and diabetes self-management across multiple LMICs could highlight population segments where public health initiatives are best focused. The primary aim of this study was to examine associations between years of education and diabetes diagnosis by education in six LMICs. In secondary analyses, associations between education and diabetes self-management behaviours among those with diabetes were considered by country.

## Methods

### Data

Data were from the first wave of the World Health Organization Study on Global Ageing and Adult Health (WHO SAGE) [17]. WHO SAGE involves nationally representative cohorts of adults aged ≥50 years from six LMICs (China, Ghana, India, Mexico, Russia, South Africa) undergoing rapid economic development. The United Nations Development Program’s (UNDP) Human Development Index (HDI) is a widely recognized tool for measuring and comparing development across countries. According to this index, the six LMICs examined in this study were considered and presented in all tables in order of development – Ghana (lowest ranking: 138 in 2010), India, South Africa, China, Mexico, Russia (ranked 60 in 2010) [18].

The response rate for the first wave ranged from 51% in Mexico to 93% in China. The dataset, as opposed to single country studies, enables systematic investigation of prevalence and socioeconomic gradients of diabetes and diabetes self-management behaviours, given the validated standardised measurement tools and approaches to data collection and management across countries. Although WHO SAGE collected data for a wave zero and some of the first wave participants featured in wave zero, this prior wave was part of the World Health Survey of 70 countries and, as such, used a different sampling approach to the WHO SAGE longitudinal cohort which began with wave one. Therefore, only the first wave of data was used for this analysis.

Full details of the study are provided elsewhere. In brief, multistage cluster random sampling was conducted in each country with all participants from households classified as ‘50+ year households’ invited to complete an individual face-to-face interview. Proxy respondents were identified for participants who could not complete the interview; these were included in the analysis where data on the key study variables were provided. Baseline interviews were conducted between 2007 and 2010. Person-level analysis weights were calculated for each country; these included both a sample selection and a post-stratification factor, with the most recent population estimates provided by the national statistical offices in each country.

### Ethics

This study uses secondary data from the WHO SAGE study which was approved by the WHO’s Ethical Review Board. Consent to use these data for this study was provided by the WHO Multi-Country Studies Data Archive.

### Diabetes

Self-reported diabetes diagnosis was recorded during face-to-face interviews, with participants asked, “Have you ever been diagnosed with diabetes (high blood sugar)?” Those who responded ‘yes’ to this question were recorded as having diabetes. Participants were asked to exclude diabetes associated with a pregnancy.

### Education

Education was used as a marker of socioeconomic status; increases in education in LMICS are believed to be an important factor in improving health [19]. Each participant was asked if they had ever been to school and how many years of schooling (including higher education) they had completed. Those who stated they had never been to school were recoded to 0 years of education.

### Diabetes self-management behaviours

Behaviours that contribute to better self-management of diabetes were considered for those who reported a diabetes diagnosis in secondary analyses.

Physical activity, defined as minutes spent in leisure-time physical activity and active transport in a typical week, and sedentary behaviour, defined as minutes spent sitting in a typical day, were recorded using the Global Physical Activity Questionnaire [20]. Fruit and vegetable consumption were both reported as the number of servings eaten on a typical day. Participants also reported if they had been following a special diet, exercise regimen, or weight control program for diabetes during the last two weeks and if they had been taking insulin or other blood sugar lowering medications during that period.

Height (cm), weight (kg), waist (cm) and hip (cm) circumferences were objectively measured by WHO SAGE interviewers. Body mass index (BMI) was calculated for each participant by dividing weight by height in metres squared (kg/m^2^).

### Other covariates

Sex, age, ethnicity and urbanicity (urban/rural) of residence of each participant were recorded. Ethnic groups varied by country (see Table 1). Ethnicity was not considered for Mexico as ethnicity was recorded as “none” or “missing” for most participants.

**Table 1:** Unweighted descriptive characteristics of the sample by country*

	Ghana	India	South Africa	China	Mexico	Russia
	***N*** = 4152	***N*** = 6505	***N*** = 3071	***N*** = 12,685	***N*** = 1954	***N*** = 3413
**Diabetes,** *n(%)*						
*Yes*	3.9%	7.2%	9.7%	6.6%	20.5%	8.6%
**Years of schooling**, *mean (SD)*	4.1 (5.3)	3.6 (4.7)	5.7 (4.7)	5.6 (4.5)	4.4 (4.1)	10.9 (3.9)
**Age (years)**, *mean (SD)*	64.3 (10.6)	61.8 (9.0)	62.7 (9.6)	63.0 (9.3)	68.6 (8.7)	64.8 (10.2)
**Gender,** *n(%)*						
*Female*	48.2%	49.6%	60.6%	53.1%	61.1%	64.5%
**Urban/rural classification,** *n(%)*						
*Urban*	40.7%	25.6%	66.5%	49.0%	71.6%	76.3%
Ethnicity**, *n(%)*	*Akan:* 49.0%	*Scheduled tribe/caste:* 22.8%	*African/Black:* 62.7%	*Han:* 98.5%	N/A	*Russian:* 80.1%
	*Ewe:* 6.9%	*No caste or tribe:* 17.2%	*White:* 7.9%	*Other:* 1.5%		*Caucasus:* 12.0%
	*Ga-Adangbe:* 10.1%	*Other:* 60.0%	*Coloured:* 20.2%			*Other:* 7.9%
	*Gruma:* 5.1%		*Indian/Asian:* 9.1%			
	*Mole-Dagbon:* 2.6%					
	*Other:* 26.3%					

*Countries are presented in order of level of development from least to most developed [17]. **No ethnicity data available for Mexico

### Statistical analysis

Survey weighted descriptive statistics were calculated for prevalence of diagnosis of diabetes for each country. To address the first aim, country-specific survey weighted log-binomial regression models were fitted to examine associations between education and diabetes diagnosis. Unadjusted analyses and analyses adjusting for potential confounding variables (age, sex, and urbanicity) were considered (Adjustment 1). Ethnicity was also considered to be a potential confounding variable. However, as data on ethnicity was not available for Mexico, ethnicity was only considered in sensitivity analyses in further adjusted models in all countries apart from Mexico (Adjustment 2), to assess whether this influenced observed associations.

To address the secondary aim, only those who reported a diabetes diagnosis were considered as the aim was to examine educational gradients in behaviours to help manage their condition among those living with diabetes. Since very few participants reported conducting any physical activity, physical activity outcomes were dichotomised. Country-specific survey-weighted logistic or log-binomial regression models were fitted to examine associations between the education and leisure time and transport physical activity (any: no/yes), special diet (no/yes), and insulin, or diabetes medication (no/yes). Linear regression was used to examine associations between education and sedentary time (square root transformed prior to modelling to address skewness), BMI, waist and hip circumference. Poisson regression was used to model associations between education and the number of servings of fruit per day; number of servings of vegetables per day; and the number of servings of fruit and vegetables per day. Analyses with and without adjustment for confounders were considered. Analyses were conducted using Stata version 14·2.

### Missing data

A complete case analysis was conducted assuming the data were missing completely at random. A small proportion of participants ranging from 0·03% in India to 4·6% in Mexico had missing diabetes data (Supplementary Table 1). Apart from South Africa (16·8%), most countries had a small proportion of missing education data. The percentage of participants with diabetes was similar among those who did (10·7%) and did not (9·7%) have missing education data. After omitting those with missing data for all covariates considered, the sample sizes were 4152 (96·5%) for Ghana, 6505 (99·2%) for India, 3071 (80·0%) for South Africa, 12,685 (96.3%) for China, 1954 (93·8%) for Mexico and 3413 (86.6%) for Russia. Comparisons of omitted and complete case samples showed complete case participants had lower average years of schooling in all countries (apart from Mexico which had no education data among those omitted), higher average age (apart from Mexico which showed the opposite) and a lower proportion of females in Ghana and India but higher in South Africa (Supplementary Table 2). Only those with complete data for all self-management behaviours among those with diabetes were considered for the second aim, resulting in sample sizes of 137 (82·0% of those with diabetes) in Ghana, 413 (86·4%) in India, 239 (66·4%) in South Africa, 654 (77.6%) in China, 315 (76·8%) in Mexico and 187 (53·4%) in Russia. Missing data was particularly high in Russia and South Africa where many participants refused to have anthropometric measurements taken. A comparison of characteristics for participants with diabetes showed the complete case sample was generally representative of the full sample (Supplementary Table 3). Although, on average, the complete case sample tended to be older than those with missing data in all countries apart from Russia.

## Results

**Fig. 1:**
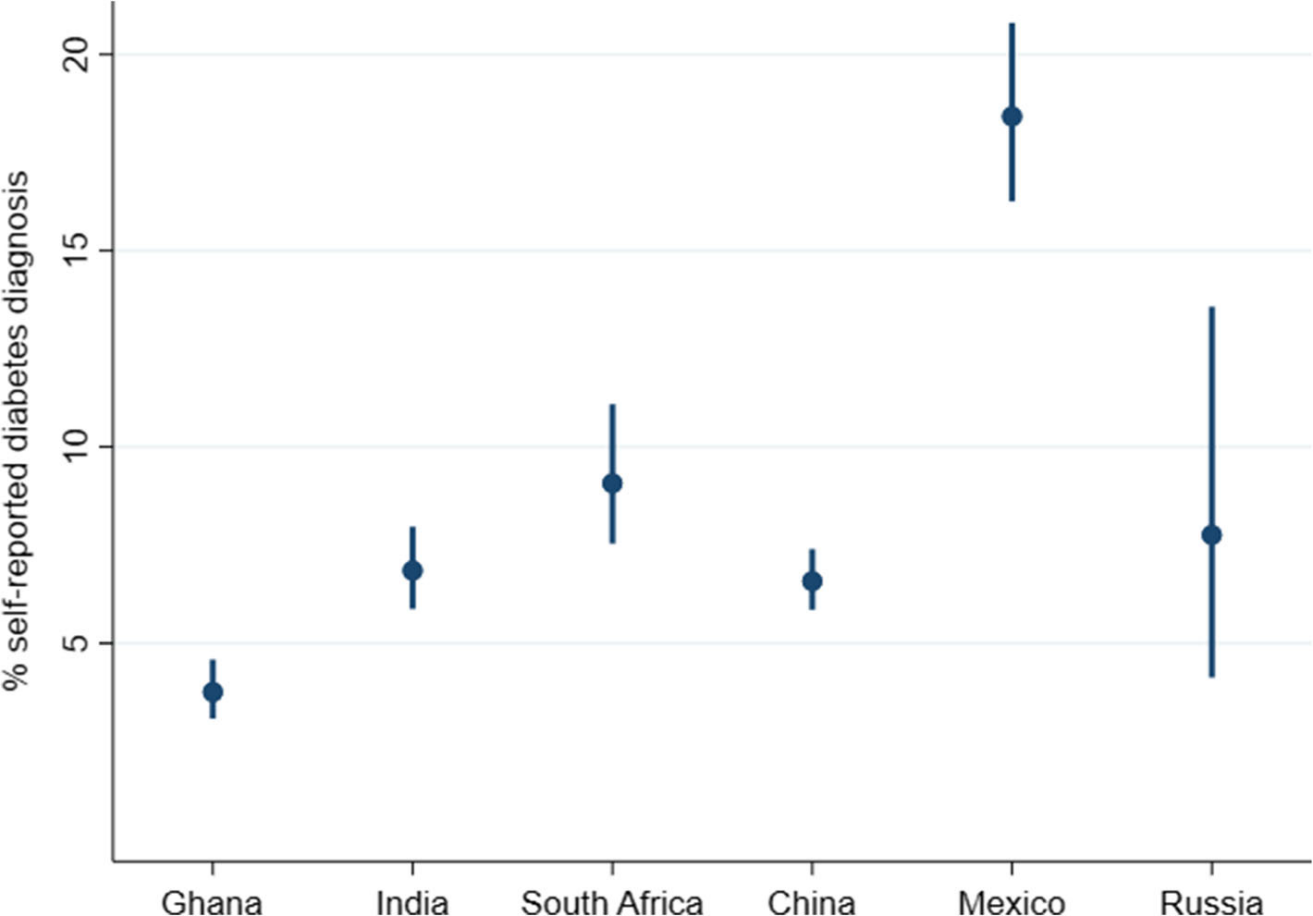
Survey-weighted-prevalence of self-reported diabetes diagnosis by country* with 95% confidence intervals. *Countries are presented in order of level of development from least to most developed [17]

The weighted prevalence of diabetes diagnosis ranged from 3·8% in Ghana to 18·4% in Mexico (Fig. 1). Unweighted descriptive characteristics for the sample are presented in Table 1. There was a lot of variability in schooling between the countries with the mean years of schooling ranging from 3.6 years (SD = 4.7) in India to 10.9 years (SD = 3.9) in Russia.

### Socioeconomic gradients in diabetes

**Fig. 2:**
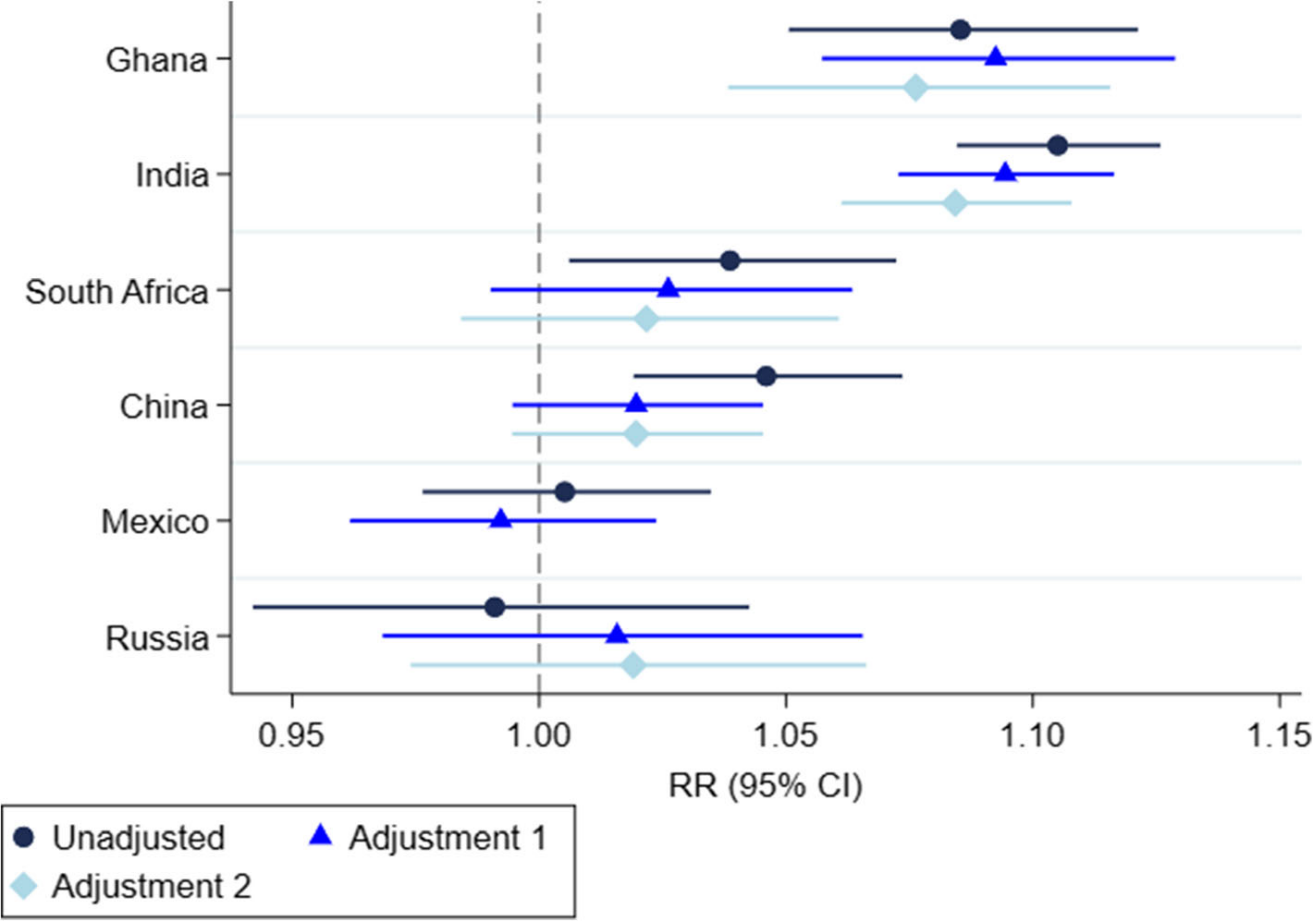
Unadjusted and adjusted risk ratio of self-reported diabetes diagnosis by education for each WHO SAGE country. *Countries are presented in order of level of development from least to most developed [17]

After accounting for confounders, a positive association between education and diabetes diagnosis was observed in Ghana (RR_adj1_ = 1.09; 95% CI = 1.06–1.13) and India (RR_adj1_ = 1.09; 95% CI = 1.07–1.12), with an estimated increase in risk of diabetes diagnosis of 9% with each additional year of education in both (Fig. 2). However, there was not strong evidence of an association between education and diabetes diagnosis for South Africa, China, Mexico, or Russia after adjustment. Estimated risk ratios are presented in Supplementary Table 4.

### Socioeconomic gradients in diabetes self-management factors among those with diagnosed diabetes

#### Physical activity and sedentary behaviour

Descriptive statistics for self-management factors for those with diabetes diagnosis are presented in Table 2. Among those with diabetes, the percentage of participants who conducted any leisure-time physical activity was low, ranging from 6.4% in Russia to 21.7% in China, but higher for any transport physical activity, ranging from 18.4% in South Africa to 70.1% in Ghana. Average fruit consumption was similar across all countries, ranging from 1.2 (SD = 0.8) in India to 2.1 (SD = 1.5) in Ghana. Average vegetable intake among participants with diabetes was highest in China (6.6 portions) and lowest in Mexico (1.6). Less than half (47.3%) of participants with diabetes in Mexico were consuming a special diet for diabetes and only approximately half (52.8%) of participants with diabetes in India were taking insulin or blood sugar lowering medication. Average body mass index among those with diabetes was highest in South Africa (31.4 kg/m^2^ [SD = 7.1]) and lowest in India (22.9 kg/m^2^ [SD = 4.4]).

**Table 2:** Unweighted sample descriptive characteristics of diabetes self-management factors by country* for those with self-reported diabetes diagnosis

	Ghana	India	South Africa	China	Mexico	Russia
	***N*** = 137	***N*** = 413	***N*** = 239	***N*** = 654	***N*** = 315	***N*** = 187
**Any leisure physical activity,** *n(%)*						
*Yes*	19.7%	19.6%	7.5%	21.7%	6.7%	6.4%
**Any transport physical activity,** *n(%)*						
*Yes*	70.1%	43.1%	18.4%	64.1%	25.7%	52.4%
**Sedentary time (mins/day),** *mean (SD)*	246.3 (147.7)	194.4 (164.2)	189.3 (138.5)	258.0 (141.4)	152.0 (122.7)	323.1 (186.5)
Fruit per day**, *mean (SD)*	2.1 (1.5)	1.2 (0.8)	1.7 (1.1)	2.0 (1.7)	1.6 (0.9)	1.4 (1.0)
Vegetables per day**, *mean (SD)*	1.9 (0.8)	2.1 (0.8)	2.1 (1.2)	6.6 (3.3)	1.6 (1.0)	1.8 (1.0)
**Special diet for diabetes,** *n(%)*						
*Yes*	70.8%	55.2%	63.6%	74.8%	47.3%	75.4%
**Insulin or blood sugar lowering medication,** *n(%)*						
*Yes*	71.5%	52.8%	87.9%	82.7%	82.9%	80.2%
**Body mass index (kg/m2)),** *mean (SD)*	25.7 (5.5)	22.9 (4.4)	31.4 (7.1)	24.9 (3.4)	28.7 (4.8)	31.3 (5.2)
**Waist circumference (cm),** *mean (SD)*	90.6 (12.7)	87.7 (10.8)	94.4 (21.4)	88.2 (9.2)	99.3 (11.3)	101.6 (13.2)
**Hip circumference (cm),** *mean (SD)*	97.5 (12.5)	93.0 (10.0)	104.4 (20.2)	97.6 (7.7)	105.6 (10.4)	110.7 (12.8)

*Countries are presented in order of level of development from least to most developed [17]. **Note: Due to extreme values for some variables, outliers (values exceeding 3 × SD from the mean) were recoded to missing for fruit, vegetables

There was moderate to strong evidence of positive associations between education and leisure-time physical activity in Ghana (OR_adj1_ = 1.15; 95% CI = 1.05–1.26) India (OR_adj1_ = 1.11; 95% CI = 1.03–1.19) and South Africa (OR_adj1_ = 1.16; 95% CI = 1.00–1.35) after adjustment, but not in the other countries (Table 3), although the association attenuates in South Africa after further adjustment for ethnicity. There was little evidence of an association between education and any transport physical activity or sedentary time for most countries after adjustment for confounders (Table 3).

**Table 3:** Associations between education and diabetes self-management factors among those with self-reported diabetes diagnosis by country

	Ghana (*N* = 137*)	India (*N* = 413*)	South Africa (*N* = 239*)	China (*N* = 654*)	Mexico (*N* = 315*)	Russia (*N* = 187)
**Leisure activity**	**OR (95% CI)**	**OR (95% CI)**	**OR (95% CI)**	**OR (95% CI)**	**OR (95% CI)**	**OR (95% CI)**
**Unadjusted**						
Education	1·16 (1·08, 1·25)	1·15 (1·08, 1·23)	1·18 (1·02, 1·37)	1·03 (0.98, 1·08)	1·09 (0·95, 1·27)	1·14 (1·04, 1·25)
	*p* < 0·001	*p* < 0·001	*p* = 0·03	*p* = 0.18	*p* = 0·21	*p* = 0.01
**Adjustment 1**						
Education	1·15 (1·05, 1·26)	1·11 (1·03, 1·19)	1·16 (1·00, 1·35)	0·97 (0·92, 1·03)	1·11 (0·96, 1·29)	1·04 (0·84, 1·28)
	*p* = 0.003	*p* = 0·01	*p* = 0·05	*p* = 0·36	*p* = 0·15	*p* = 0·74
**Adjustment 2**						
Education	1·15 (1·05, 1·26)	1·11 (1·03, 1·21)	1·07 (0·88, 1·30)	0·97 (0·92, 1·03)	–	1.05 (0.86, 1.27)
	*p* = 0.002	*p* = 0·01	*p* = 0·48	*p* = 0·34		*p* = 0.65
**Transport activity**	**OR (95% CI)**	**OR (95% CI)**	**OR (95% CI)**	**OR (95% CI)**	**OR (95% CI)**	**OR (95% CI)**
**Unadjusted**						
Education	1·06 (0.99, 1·13)	1·00 (0·94, 1·05)	1·03 (0·91, 1·17)	1·02 (0·98, 1·06)	0·95 (0·86, 1·05)	1·17 (1·03, 1·32)
	*p* = 0·08	*p* = 0·88	*p* = 0·61	*p* = 0·33	*p* = 0·31	*p* = 0·02
**Adjustment 1**						
Education	1·03 (0·95, 1·12)	1·00 (0·95, 1·07)	1·03 (0·91, 1·17)	0·97 (0·93, 1·01)	0·90 (0·82, 0·99)	1·10 (0·96, 1·26)
	*p* = 0·44	*p* = 0·77	*p* = 0·61	*p* = 0·14	*p* = 0·03	*p* = 0·15
**Adjustment 2**						
Education	1·03 (0·94, 1·13)	1·01 (0·95, 1·08)	1·02 (0·92, 1·13)	0·97 (0·92, 1·01)	–	1·07 (0·94, 1·23)
	*p* = 0·53	*p* = 0·70	*p* = 0·74	*p* = 0·13		*p* = 0·30
√(**Sedentary time) (mins/day)**	**β (95% CI)**	**β (95% CI)**	**β (95% CI)**	**β (95% CI)**	**β (95% CI)**	**β (95% CI)**
**Unadjusted**						
Education	−0·04 (−0·16, 0·08)	0·01 (−0·15, 0·18)	−0·004 (−0·24, 0·24)	−0·09 (−0·19, 0·02)	0·09 (−0·09, 0·27)	−0·03 (−0·36, 0·31)
	*p* = 0·52	*p* = 0·87	*p* = 0·98	*p* = 0·10	*p* = 0·34	*p* = 0·88
**Adjustment 1**						
Education	0·04 (−0·11, 0·19)	0·02 (−0·21, 0·24)	0·001 (−0·28, 0·28)	0·01 (−0·10, 0·11)	0·07 (−0·11, 0·25)	0·06 (−0·40, 0·52)
	*p* = 0·56	*p* = 0·90	*p* = 1·00	*p* = 0·90	*p* = 0·44	*p* = 0·79
**Adjustment 2**						
Education	0·05 (−0·10, 0·19)	0.01 (−0·21, 0·24)	−0·07 (−0·35, 0·21)	0·01 (−0·10, 0·11)	–	0·08 (−0·31, 0·47)
	*p* = 0·52	*p* = 0·90	*p* = 0·62	*p* = 0·89		*p* = 0·68
**Fruit**	**RR (95% CI)**	**RR (95% CI)**	**RR (95% CI)**	**RR (95% CI)**	**RR (95% CI)**	**RR (95% CI)**
**Unadjusted**						
Education	1·01 (0·99, 1·02)	1·02 (1·00, 1·04)	1·02 (0·98, 1·05)	1·03 (1·02, 1·05)	1·02 (1·00, 1·05)	1·04 (0·98, 1·10)
	*p* = 0·38	*p* = 0·02	*p* = 0·34	*p* < 0·001	*p* = 0·05	*p* = 0·24
**Adjustment 1**						
Education	1·02 (0·99, 1·04)	1·02 (1·00, 1·04)	1·01 (0·98, 1·05)	1·03 (1·02, 1·05)	1·02 (1·00, 1·04)	1·04 (0·99, 1·10)
	*p* = 0·23	*p* = 0·10	*p* = 0·50	*p* < 0·001	*p* = 0·12	*p* = 0·13
**Adjustment 2**						
Education	1·02 (0·99, 1·04)	1·02 (1·00, 1·04)	1·00 (0·97, 1·03)	1·03 (1·02, 1·05)	–	1·03 (0·99, 1·07)
	*p* = 0·21	*p* = 0·11	*p* = 0·99	*p* < 0·001		*p* = 0·19
**Vegetables**	**RR (95% CI)**	**RR (95% CI)**	**RR (95% CI)**	**RR (95% CI)**	**RR (95% CI)**	**RR (95% CI)**
**Unadjusted**						
Education	1·00 (0·99, 1·01)	1·01 (1·00, 1·02)	1·01 (0·98, 1·04)	1·00 (0·99, 1·01)	1·02 (1·00, 1·04)	1·04 (1·01, 1·08)
	*p* = 0·52	*p* = 0·01	*p* = 0·66	*p* = 0·93	*p* = 0·06	*p* = 0·02
**Adjustment 1**						
Education	1·00 (0·99, 1·02)	1·01 (1·00, 1·02)	1·01 (0·98, 1·04)	1·00 (0.99, 1·01)	1·02 (1·00, 1·04)	1·05 (1·02, 1·07)
	*p* = 0·52	*p* = 0·04	*p* = 0·42	*p* = 0·41	*p* = 0·13	*p* < 0·001
**Adjustment 2**						
Education	1·00 (0·99, 1·01)	1·01 (1·00, 1·02)	1·00 (0·98, 1·03)	1·00 (0.99, 1·01)	–	1·04 (1·02, 1·06)
	*p* = 0·58	*p* = 0·06	*p* = 0·72	*p* = 0·49		*p* < 0·001
**Fruit and vegetables**	**RR (95% CI)**	**RR (95% CI)**	**RR (95% CI)**	**RR (95% CI)**	**RR (95% CI)**	**RR (95% CI)**
**Unadjusted**						
Education	1·00 (0·99, 1·02)	1·02 (1·01, 1·03)	1·01 (0·98, 1·04)	1·01 (1·00, 1·02)	1·02 (1·00, 1·04)	1·04 (1·00, 1·08)
	*p* = 0·39	*p* = 0·003	*p* = 0·49	*p* = 0·05	*p* = 0·01	*p* = 0·07
**Adjustment 1**						
Education	1·01 (0·99, 1·03)	1·01 (1·00, 1·02)	1·01 (0·98, 1·04)	1·01 (1·00, 1·02)	1·02 (1·00, 1·04)	1·04 (1·01, 1·08)
	*p* = 0·28	*p* = 0·02	*p* = 0·43	*p* = 0·03	*p* = 0·04	*p* = 0·01
**Adjustment 2**						
Education	1·01 (0·99, 1·03)	1·01 (1·00, 1·02)	1·00 (0·98, 1·03)	1·01 (1·00, 1·02)	–	1·03 (1·01, 1·06)
	*p* = 0·27	*p* = 0·03	*p* = 0·83	*p* = 0·04		*p* = 0·01
**Special diet**	**RR (95% CI)**	**RR (95% CI)**	**RR (95% CI)**	**RR (95% CI)**	**RR (95% CI)**	**RR (95% CI)**
**Unadjusted**						
Education	1·00 (0·98, 1·02)**	1·04 (1·01, 1·06)**	1·00 (0·97, 1·02)**	1·00 (0·98, 1·01)**	1·03 (1·00, 1·06)**	0·98 (0·96, 1·01)**
	*p* = 0·84	*p* = 0·003	*p* = 0·83	*p* = 0·51	*p* = 0·11	*p* = 0·16
**Adjustment 1**						
Education	1·00 (0·98, 1·02)**	1·06 (1·02, 1·09)**	1·01 (0·97, 1·04)**	0·99 (0·97, 1·01)**	1·03 (1·00, 1·06)**	0·98 (0·94, 1·02)**
	*p* = 0·84	*p* < 0·001	*p* = 0·63	*p* = 0·30	*p* = 0·13	*p* = 0·32
**Adjustment 2**						
Education	0·99 (0·97, 1·02)**	1·06 (1·03, 1·09)**	0·99 (0·96, 1·03)**	0·99 (0·97, 1·01)**	**–**	0·98 (0·95, 1·02)**
	*p* = 0·64	*p* < 0·001	*p* = 0·67	*p* = 0·29		*p* = 0·34
**Insulin/blood sugar lowering medication**	**RR (95% CI)**	**RR (95% CI)**	**RR (95% CI)**	**RR (95% CI)**	**RR (95% CI)**	**RR (95% CI)**
**Unadjusted**						
Education	1·01 (0·99, 1·02)	1·03 (1·01, 1·06)	1·00 (1·00, 1·01)**	1·00 (0·99, 1·01)**	1·01 (1·00, 1·03)**	0·97 (0·91, 0·98)**
	*p* = 0·54	*p* = 0·01	*p* = 0·31	*p* = 0·62	*p* = 0·18	*p* = 0·003
**Adjustment 1**						
Education	1·00 (0·99, 1·02)	1·04 (1·01, 1·07)**	1·01 (1·00, 1·02)**	1·00 (0·99, 1·01)**	1·01 (0·99, 1·02)**	0·94 (0·91, 0·98)**
	*p* = 0·77	*p* = 0·02	*p* = 0·25	*p* = 0·78	*p* = 0·32	*p* = 0·001
**Adjustment 2**						
Education	1·00 (0·98, 1·02)	1·04 (1·01, 1·07)**	1·00 (0·99, 1·01)**	1·00 (0·99, 1·02)**	**–**	0·96 (0·92, 0·99)**
	*p* = 0·99	*p* = 0·02	*p* = 0·75	*p* = 0·64		*p* = 0·01
**Body mass index (kg/m**^**2**^**)**	**β (95% CI)**	**β (95% CI)**	**β (95% CI)**	**β (95% CI)**	**β (95% CI)**	**β (95% CI)**
**Unadjusted**						
Education	0·19 (0·02, 0·36)	0·11 (0·01, 0·21)	0·16 (−0·22, 0·55)	−0·07 (−0·14, −0·004)	−0·02 (−0·19, 0·16)	−0·08 (−0·35, 0·20)
	*p* = 0·026	*p* = 0·03	*p* = 0·41	*p* = 0·04	*p* = 0·86	*p* = 0·59
**Adjustment 1**						
Education	0·16 (−0·04, 0·36)	0·22 (0·11, 0·34)	0·16 (−0·23, 0·55)	−0·09 (−0·17, −0·02)	−0·02 (−0·18, 0·14)	−0·04 (−0·26, 0·18)
	*p* = 0·12	*p* < 0·001	*p* = 0·43	*p* = 0·01	*p* = 0·81	*p* = 0·72
**Adjustment 2**						
Education	0·11 (−0·07, 0·28)	0·22 (0·10, 0·33)	0·31 (−0·04, 0·67)	−0·10 (−0·17, −0·03)	–	0·02 (−0·20, 0·24)
	*p* = 0·23	*p* < 0·001	*p* = 0·08	*p* = 0·005		*p* = 0·86
**Waist circumference (cm)**	**β (95% CI)**	**β (95% CI)**	**β (95% CI)**	**β (95% CI)**	**β (95% CI)**	**β (95% CI)**
**Unadjusted**						
Education	0·21 (−0·13, 0·55)	0·52 (0·25, 0·79)	0·09 (−0·78, 0·95)	−0·05 (−0·27, 0·17)	0·20 (−0·29, 0·68)	−0·05 (−0·73, 0·62)
	*p* = 0·23	*p* < 0·001	*p* = 0·84	*p* = 0·65	*p* = 0·42	*p* = 0·87
**Adjustment 1**						
Education	0·23 (−0·24, 0·70)	0·71 (0·39, 1·03)	0·05 (−0·86, 0·96)	−0·12 (−0·31, 0·07)	0·12 (−0·32, 0·57)	−0·01 (−0·54, 0·52)
	*p* = 0·33	*p* < 0·001	*p* = 0·91	*p* = 0·20	*p* = 0·59	*p* = 0·98
**Adjustment 2**						
Education	0·17 (−0·24, 0·59)	0·69 (0·36, 1·01)	0·19 (−0·64, 1·02)	−0·13 (−0·31, 0·05)		0·14 (−0·47, 0·75)
	*p* = 0·41	*p* < 0·001	*p* = 0·65	*p* = 0·15	–	*p* = 0·65
**Hip circumference (cm)**	**β (95% CI)**	**β (95% CI)**	**β (95% CI)**	**β (95% CI)**	**β (95% CI)**	**β (95% CI)**
**Unadjusted**						
Education	0·31 (−0·07, 0·68)	0·11 (−0·09, 0·31)	−0·33 (−1·12, 0·45)	0·10 (−0·11, 0·30)	0·15 (−0·27, 0·57)	−0·22 (−0·98, 0·55)
	*p* = 0·11	*p* = 0·27	*p* = 0·40	*p* = 0·35	*p* = 0·49	*p* = 0·57
**Adjustment 1**						
Education	0·33 (−0·17, 0·82)	0·38 (0·18, 0·58)	−0·38 (−1·24, 0·48)	0·12 (−0·10, 0·34)	0·23 (−0·20, 0·66)	0·11 (−0·54, 0·76)
	*p* = 0·19	*p* < 0·001	*p* = 0·39	*p* = 0·29	*p* = 0·29	*p* = 0·73
**Adjustment 2**						
Education	0·26 (−0·18, 0·70)	0·35 (0·15, 0·55)	−0·16 (−0·87, 0·56)	0·11 (−0·10, 0·33)	–	0·23 (−0·40, 0·87)
	*p* = 0·25	*p* = 0·001	*p* = 0·67	*p* = 0·29		*p* = 0·47

*Countries are presented in order of level of development from least to most developed [17]. ^1^Analysis adjusts for survey weights. ^*^Number of participants in the sample who provided complete data for variables in analysis. ^**^Poisson regression used to obtain approximate risk ratios as log-binomial regression failed to converge. Adjustment 1: models adjusted for age, sex and urbanicity. Adjustment 2: models adjusted for age, sex, urbanicity and ethnicity

#### Fruit and vegetable intake

Although point estimates for the associations between education and fruit, vegetable, or fruit and vegetable intake combined were all in the same direction for the six countries (Table 3), suggesting increased consumption among those with higher education, estimated risk ratios were low (1.00–1.04). Furthermore, there was only strong evidence of an association between education and fruit intake in China, education and vegetable intake in India and Russia, and education and fruit and vegetable intake combined in China, India, Mexico, and Russia.

#### Special diet and medication

There was only weak evidence of an association between education and the likelihood of being on a special diet among those with diabetes for most countries apart from India where the rates of being on a special diet (RR_adj1_ = 1.06; 95% CI = 1.02–1.09) or of taking insulin or diabetes medication (RR_adj1_ = 1.04; 95% CI = 1.01–1.07) increased with increasing years of education. The opposite pattern for medication was observed in Russia (RR_adj1_ = 0.94; 95% CI = 0.91–0.98).

#### BMI, waist circumference and hip circumference

Associations between education and the adiposity measures (BMI, waist circumference, and hip circumference) among participants with diabetes diagnosis were inconsistent across countries (Table 3). There was only evidence of an association between education and all three outcomes in India, with higher education found to be associated with higher average BMI, waist circumference and hip circumference.

## Discussion

Our study provides evidence on associations between years of education and self-reported diabetes diagnosis and diabetes self-management behaviours in six LMICs undergoing rapid economic development. Results showed that while self-reported diabetes diagnosis was more prevalent amongst the most educated in Ghana and India – the least developed countries examined – there was no evidence of an association between years of education and diabetes in Russia or Mexico, or in China or South Africa after confounder adjustment.

Our study advances limited existing evidence on the relationship between socioeconomic status and diabetes in LMICs [13]. Findings are broadly consistent with those of prior reports [21–23] on the existence and direction of associations of education with diabetes in individual countries. For example, prior research from Chinese adults aged over 45 years found no evidence of an association between education and diabetes consistent with our findings [21], as did research from Russia [23]. Furthermore, among older adults in India, higher education was associated with higher numbers of self-reported diagnosed chronic diseases, consistent with our results [22]. In addition, a systematic review of predictors of diabetes diagnosis in Ghana highlighted secondary and tertiary level of education as a risk factor for diabetes in some prior research, in line with our findings [24]. In contrast to our findings, prior longitudinal research from Mexico showed higher education was associated with a lower probability of diabetes diagnosis when participants were asked if a doctor or medical personnel had ever told them that they had diabetes or a high blood sugar level [25]. Reasons for inconsistent findings could include variations in the populations, sampling frames or methods used to assess education and diabetes. Our study advances this work by comparing multiple countries using standardised approaches.

The positive association between education and self-reported diabetes diagnosis in Ghana and India – the two least developed countries included in this study – may reflect the greater accessibility of desirable westernised practices, such as vehicle ownership and sedentary lifestyles, and increased fast food, soft drink and alcohol consumption, amongst those of a higher educational status [26–28]. These groups may have greater means of engaging in these behaviours which place them at increased risk of overweight and diabetes. That these associations were not observed in the remaining countries studied may be attributable to their more advanced levels of development. Economic development in countries results in resolution of food shortage problems, even among the poor; increasing availability of energy-dense foods including chain brand fast food outlets; and increased automation and reduced manual labour in workplaces and communities [29]. Such changes plausibly play a role in the rise of NCDs like diabetes through their impact on population diet and physical activity levels. Against the backdrop of these changes, the shifting distribution of diabetes risk away from the most advantaged is consistent with explanations stemming from diffusion of innovations theory [30, 31], whereby higher educated individuals tend to be the first to become aware of new knowledge (such as disease risk factors), and quicker to adapt behaviours to reduce risk.

It is importantly to acknowledge that the WHO SAGE study relied on self-report of diabetes diagnosis. Therefore, it is possible that differences observed between countries may be due to under-reporting of diabetes across the different contexts. It is probable that there is a relationship between education and the likelihood of seeking care and receiving a diagnosis for diabetes. For example, research from South Africa found socioeconomic inequalities in diabetes, measured using wealth indictors, with higher levels of self-reported diabetes diagnosis among the wealthier who have greater access to health care [32]. Furthermore, the prevalence of diabetes diagnosis reported appeared high in some contexts leading to questions about the representativeness of all samples. While the estimated prevalence was within the 95% confidence intervals for 2010 estimates from a pooled analysis of 751 population-based studies for almost all of the countries [33], the estimated prevalence of almost 20% in Mexico was found to be high compared to the estimated 10.4% (95% CI: 5.9–14.8%) in this study, although some data sources used in that analysis spanned a greater age distribution than in WHO SAGE, with adults as young as 20 years included.

Acknowledging the cross-sectional design and challenges with self-reported diabetes diagnosis, the findings according to country level of development are consistent with other evidence of shifting socioeconomic gradients in NCDs as countries transition to higher income [10, 29, 34, 35]. While we cannot determine trends from the present study, should the countries examined here follow these patterns, without intervention the risk of diabetes may soon shift to the most disadvantaged, with the more developed countries here possibly in the midst of this shift, and Ghana and India (depending on future rates of development) potentially the last to experience this gradient reversal.

Diabetes self-management is important since it can help patients enhance diabetes control and significantly reduce their likelihood of long-term complications [36]. However, data regarding diabetes self-management in LMICs are sparse. While rates of engagement in self-management behaviours varied greatly across countries and behaviours, in many cases these were less than optimal. A limited number of previous studies have examined medication compliance or other individual behaviours amongst people with diabetes in these contexts [37–39], but to our knowledge this study is the first to systematically investigate the engagement in behaviours that aid in diabetes self-management across multiple LMICs, and the distribution of these behaviours according to education among people with diabetes. Findings from WHO SAGE showed that among people self-reporting diabetes diagnosis, higher education was associated with more leisure-time physical activity in Ghana and India only. The opportunity to engage in leisure-time physical activity opportunities may be more restricted amongst the least educated in these countries. Other studies in LMICs have shown similar associations [26, 40]. We found no evidence of educational gradients in leisure-time physical activity in other countries. This may be attributable to relatively low proportions of people with diabetes reporting any leisure-time physical activity (from only ~ 6·0% in Mexico and South Africa, to 21·5% in China), resulting in insufficient variability to detect strong gradients. We found no evidence of associations between education and either transport-related activity or sedentary behaviours among people with diabetes in any country. Technological progress across all countries may have resulted in widespread access to both automated work systems and sedentary pursuits across educational groups [41, 42]. The somewhat mixed nature of observed associations of education level with these and the remaining behavioural factors examined is in line with findings of a systematic review of the socioeconomic gradients in NCD behavioural risk factors in LMICs [40]. That review concluded that despite some evidence of socioeconomic gradients, heterogeneity between measures limited the certainty of their findings, noting that studies of the distribution of behavioural risk factors in these countries are scarce and produce inconsistent results.

Strengths of this study include the large, multi-country sample, and standardised sampling and measurement approaches applied systematically across all countries to ensure comparability and the ability to adjust for key potential confounders. The sample with diabetes was recruited from the population, and not only limited to those attending clinics or known to medical or community workers. Limitations include the self-reported measure of diabetes, which may have resulted in exclusion of unknown or undiagnosed cases and an underestimation of prevalence. This may be particularly pronounced in low socioeconomic status groups who have less access to healthcare and could lead to false positive associations of socioeconomic position with disease [7]. We could not distinguish Type 1 and Type 2 diabetes. Furthermore, medication and insulin prescription and use may vary depending on unmeasured factors, such as disease severity. Aside from taking insulin/medication or being on a special diet, we also could not examine whether individuals engaging in behavioural factors did so specifically in order to help manage their diabetes.

## Conclusions

Efforts to manage diabetes in low-resource countries are hindered by a lack of rigorous data on risk groups and behaviours. Our findings help address this gap and have significance for governments, policy makers and health workers. They provide robust data on the distribution of diabetes and its behavioural self-management in LMICs that can assist in identifying priority groups for targeted education and behavioural support for diabetes control. While diabetes remains a disease disproportionately affecting the higher educated in least developed LMICs, this is not the case in countries undergoing rapid economic development, where no such associations were apparent. In the context of limited existing data, and considering typical trends by which increasing development shifts the socioeconomic gradients in diabetes risk towards the poor, our findings highlight the urgent need for enhanced awareness and improvement of disease protective factors, particularly amongst those with low education in these countries.

## Supplementary Information

**Additional file 1.**

## Data Availability

Data for this study were from the World Health Organization Study on global AGEing and adult health (SAGE). The de-identified data and meta-data are freely available by request through the WHO SAGE website: https://apps.who.int/healthinfo/systems/surveydata/index.php/catalog/sage.

## Abbreviations

LMIC: Low and middle income country
HIC: High income country
GDP: Gross domestic product
NCD: Non-communicable disease
WHO SAGE: World Health Organization Study on Global Ageing and Adult Health
UNDP: United Nations Development Program
HDI: Human Development Index
BMI: Body Mass Index
SD: Standard Deviation
CI: Confidence Interval
RR: Risk ratio
OR: Odds ratio
SDG: Sustainable Development Goal
