# Supplementary material for: Educational differences in diabetes and diabetes self-management behaviours in WHO SAGE countries
Source: BMC Public Health. 2021 Nov 17;21:2108. doi: 10.1186/s12889-021-12131-7 (PMC8597224; doi:10.1186/s12889-021-12131-7)
Supplement: Supplementary file 1 — Additional file 1. [file 12889_2021_12131_MOESM1_ESM.docx]

**Supplementary Table 1.** Unweighted descriptive characteristics of the sample by country.

|  | **Ghana**  **(N = 4305)** | **India**  **(N = 6560)** | **South Africa**  **(N = 3838)** | **China**  **(N = 13175)** | **Mexico**  **(N = 2083)** | **Russia**  **(N = 3938)** |
| --- | --- | --- | --- | --- | --- | --- |
| **Diabetes, n (%)**  *No*  *Yes*  *Missing* | 4122 (95·8%)  167 (3·9%)  16 (0·4%) | 6080 (92·7%)  478 (7·3%)  2 (0·03%) | 3300 (86·0%)  360 (9·4%)  178 (4·6%) | 12066 (91·6%)  843 (6·4%)  266 (2·0%) | 1579 (75·8%)  410 (19·7%)  94 (4·5%) | 3532 (89·7%)  350 (8·9%)  56 (1·4%) |
| **Education (years)**  *Mean (SD)*  *Min – Max*  *Missing, n (%)* | 4·1 (5·3)  0 – 26  65 (1·5%) | 3·6 (4·7)  0 – 23  20 (0·3%) | 5·8 (4·7)  0 – 26  644 (16·8%) | 5·5 (4·5)  0 – 23  178 (1·4%) | 4·4 (4·1)  0 – 25  129 (6·2%) | 10·9 (3·9)  0 – 25  104 (2·6%) |
| **Age (years)**  *Mean (SD)*  *Min – Max* | 64·3 (10·7)  50 – 114 | 61·8 (9·0)  50 – 106 | 62·7 (9·7)  50 – 113 | 63·2 (9·4)  50 – 99 | 69·2 (9·1)  50 – 105 | 65·1 (10·2)  50 – 100 |
| **Sex, n (%)**  *Male*  *Female* | 2249 (52·2%)  2056 (47·8%) | 3304 (50·4%)  3256 (49·6%) | 1636 (42·6%)  2202 (57·4%) | 6171 (46·8%)  7004 (53·2%) | 811 (38·9%)  1272 (61·1%) | 1395 (35·4%)  2543 (64·6%) |
| **Ethnicity, n (%)** | Akan: 2061 (47·9%)  Ewe: 291 (6·8%)  Ga-Adangbe: 437 (10·2%)  Gruma: 215 (5·0%)  Mole-Dagbon: 107 (2·5%)  Other: 1116 (25·9%)  Missing: 78 (1·8%) | Scheduled tribe/caste: 1485 (22·6%)  No tribe/caste: 1124 (17·1%)  Other: 3918 (59·7%)  Missing: 33 (0·5%) | African/Black: 2053 (53·5%)  White: 268 (7·0%)  Coloured: 655 (17·1%)  Indian/Asian: 307 (8·0%)  Missing: 555 (14·5%) | Han: 12740 (96·7%)  Other: 198 (1·5%)  Missing: 237 (1·8%) | N/A* | Russian: 2979 (75·7%)  Other: 286 (7·3%)  Caucasus: 451 (11·5%)  Missing: 222 (5·6%) |
| **Urbanicity, n (%)**  *Urban*  *Rural*  *Missing* | 1761 (40·9%)  2544 (59·1%)  0 (0·0%) | 1676 (25·6%)  4884 (74·5%)  0 (0·0%) | 2559 (66·7%)  1276 (33·3%) | 6428 (48·8%)  6747 (51·2%)  0 (0·0%) | 1501 (72·1%)  582 (27·9%) | 3003 (76·3%)  935 (23·7%) |

*No ethnicity data available for Mexico.

**Supplementary Table 2.** Unweighted descriptive characteristics of the full, complete case and omitted sample of participants by country.

|  |  | **Ghana** |  |  |  | **India** |  |  |  | **South Africa** |  |  |
| --- | --- | --- | --- | --- | --- | --- | --- | --- | --- | --- | --- | --- |
|  | **Full sample** | **Omitted** | **Complete case** | **p*** | **Full sample** | **Omitted** | **Complete case** | **p*** | **Full sample** | **Omitted** | **Complete case** | **p*** |
|  | 5573 | 1421 | 4152 |  | 12198 | 5693 | 6505 |  | 4227 | 1156 | 3071 |  |
| Diabetes |  |  |  |  |  |  |  |  |  |  |  |  |
| *Yes* | 3.5% | 1.8% | 3.9% | 0.002 | 4.9% | 1.7% | 7.2% | <0.001 | 9.2% | 7.5% | 9.7% | 0.042 |
| Years of schooling, mean (SD) | 4.5 (5.4) | 6.2 (5.2) | 4.1 (5.3) | <0.001 | 4.3 (5.0) | 5.3 (5.1) | 3.6 (4.7) | <0.001 | 6.0 (4.8) | 8.4 (4.8) | 5.7 (4.7) | <0.001 |
| Age (years), mean (SD) | 60.2 (14.1) | 47.9 (16.0) | 64.3 (10.6) | <0.001 | 50.0 (16.8) | 36.4 (12.9) | 61.8 (9.0) | <0.001 | 60.3 (12.4) | 53.8 (16.0) | 62.7 (9.6) | <0.001 |
| Gender |  |  |  |  |  |  |  |  |  |  |  | <0.001 |
| *Female* | 49.4% | 52.9% | 48.2% | 0.003 | 61.4% | 74.8% | 49.6% | <0.001 | 57.5% | 49.2% | 60.6% |  |
| Urban/rural classification |  |  |  |  |  |  |  |  |  |  |  |  |
| *Urban* | 40.9% | 41.8% | 40.7% | 0.45 | 25.7% | 25.8% | 25.6% | 0.80 | 66.6% | 66.9% | 66.5% | 0.80 |
| Ethnicity |  |  |  |  |  |  |  |  |  |  |  |  |
|  | Akan=49.1% | 49.5% | 49.0% | 0.23 | Scheduled tribe/caste=24.7% | 27.3% | 22.8% | <0.001 | African/Black=62.6% | 61.8% | 62.7% | 0.55 |
|  | Ewe=6.6% | 5.2% | 6.9% |  | No caste or tribe=17.1% | 16.9% | 17.2% |  | White=8.0% | 8.5% | 7.9% |  |
|  | Ga-Adangbe=10.5% | 12.3% | 10.1% |  | Other=58.2% | 55.8% | 60.0% |  | Coloured=20.0% | 18.8% | 20.2% |  |
|  | Gruma=5.1% | 5.1% | 5.1% |  |  |  |  |  | Indian/Asian=9.4% | 10.9% | 9.1% |  |
|  | Other=26.2% | 25.6% | 26.3% |  |  |  |  |  |  |  |  |  |
|  | Mole-Dagbon=2.5% | 2.3% | 2.6% |  |  |  |  |  |  |  |  |  |

|  |  | **China** |  |  |  | **Mexico** |  |  |  | **Russia** |  |  |
| --- | --- | --- | --- | --- | --- | --- | --- | --- | --- | --- | --- | --- |
|  | **Full sample** | **Omitted** | **Complete case** | **p*** | **Full sample** | **Omitted** | **Complete case** | **p*** | **Full sample** | **Omitted** | **Complete case** | **p*** |
|  | 15050 | 2365 | 12685 |  | 2083 | 129 | 1954 |  | 4947 | 1534 | 3413 |  |
| Diabetes |  |  |  |  |  |  |  |  |  |  |  |  |
| *Yes* | 6.0% | 2.0% | 6.6% | <0.001 | 20.6% | 25.7% | 20.5% | 0.45 | 8.3% | 7.0% | 8.6% | 0.12 |
| Years of schooling, mean (SD) | 5.8 (4.6) | 7.0 (5.0) | 5.6 (4.5) | <0.001 | 4.4 (4.1) | N/A | 4.4 (4.1) | N/A | 11.1 (3.8) | 11.9 (3.5) | 10.9 (3.9) | <0.001 |
| Age (years), mean (SD) | 60.5 (11.9) | 47.0 (15.1) | 63.0 (9.3) | <0.001 | 69.2 (9.1) | 77.8 (10.8) | 68.6 (8.7) | <0.001 | 62.3 (13.0) | 53.4 (17.6) | 64.8 (10.2) | <0.001 |
| Gender |  |  |  |  |  |  |  |  |  |  |  |  |
| *Female* | 53.4% | 54.9% | 53.1% | 0.12 | 61.1% | 61.2% | 61.1% | 0.97 | 64.4% | 64.0% | 64.5% | 0.76 |
| Urban/rural classification |  |  |  |  |  |  |  |  |  |  |  |  |
| *Urban* | 49.0% | 49.1% | 49.0% | 0.94 | 72.1% | 78.3% | 71.6% | 0.10 | 75.0% | 72.0% | 76.3% | 0.001 |
| Ethnicity |  |  |  |  |  |  |  |  |  |  |  |  |
|  | Han=98.5% | 98.4% | 98.5% | 0.80 |  |  |  |  | Russian=79.5% | 76.9% | 80.1% | 0.025 |
|  | Other=1.5% | 1.6% | 1.5% |  |  |  |  |  | Other=7.9% | 7.4% | 7.9% |  |
|  |  |  |  |  |  |  |  |  | Caucasus=12.6% | 15.7% | 12.0% |  |

**Supplementary Table 3.** Unweighted descriptive characteristics of the full, complete case and omitted sample of participants with self-reported diabetes diagnosis by country.

|  |  | **Ghana** |  |  |  | **India** |  |  |  | **South Africa** | |  |
| --- | --- | --- | --- | --- | --- | --- | --- | --- | --- | --- | --- | --- |
|  | **Full sample** | **Omitted** | **Complete case** | **p*** | **Full sample** | **Omitted** | **Complete case** | **p*** | **Full sample** | **Omitted** | **Complete case** | **p*** |
|  | 177 | 40 | 137 |  | 550 | 137 | 413 |  | 370 | 131 | 239 |  |
| Any leisure-time physical activity |  |  |  |  |  |  |  |  |  |  |  |  |
| *Yes* | 29 (16.8%) | 2 (5.6%) | 27 (19.7%) | 0.043 | 107 (20.0%) | 26 (21.1%) | 81 (19.6%) | 0.71 | 25 (7.0%) | 7 (6.0%) | 18 (7.5%) | 0.59 |
| Any transport physical activity |  |  |  |  |  |  |  |  |  |  |  |  |
| *Yes* | 115 (66.5%) | 19 (52.8%) | 96 (70.1%) | 0.050 | 237 (44.2%) | 59 (48.0%) | 178 (43.1%) | 0.34 | 65 (18.3%) | 21 (17.9%) | 44 (18.4%) | 0.92 |
| Sedentary time (mins), mean (SD) | 253.5 (153.4) | 278.1 (171.1) | 246.3 (147.7) | 0.25 | 191.2 (159.6) | 181.7 (144.9) | 194.4 (164.2) | 0.42 | 187.6 (137.5) | 184.5 (136.0) | 189.3 (138.5) | 0.75 |
| Fruit intake, mean (SD) | 2.2 (1.5) | 2.4 (1.5) | 2.1 (1.5) | 0.22 | 1.1 (0.8) | 0.9 (0.9) | 1.2 (0.8) | 0.002 | 1.7 (1.1) | 1.7 (1.2) | 1.7 (1.1) | 0.46 |
| Vegetable intake, mean (SD) | 1.9 (0.9) | 2.2 (0.9) | 1.9 (0.8) | 0.072 | 2.1 (0.8) | 2.0 (0.8) | 2.1 (0.8) | 0.73 | 2.2 (1.2) | 2.3 (1.3) | 2.1 (1.2) | 0.28 |
| Special diet for diabetes |  |  |  |  |  |  |  |  |  |  |  |  |
| *Yes* | 122 (69.3%) | 25 (64.1%) | 97 (70.8%) | 0.42 | 303 (55.1%) | 75 (54.7%) | 228 (55.2%) | 0.93 | 239 (64.9%) | 87 (67.4%) | 152 (63.6%) | 0.46 |
| Insulin or blood sugar lowering medication |  |  |  |  |  |  |  |  |  |  |  |  |
| *Yes* | 128 (72.3%) | 30 (75.0%) | 98 (71.5%) | 0.67 | 289 (52.5%) | 71 (51.8%) | 218 (52.8%) | 0.85 | 316 (85.6%) | 106 (81.5%) | 210 (87.9%) | 0.098 |
| Body mass index (kg/m2)), mean (SD) | 25.7 (5.4) | 25.8 (5.0) | 25.7 (5.5) | 0.95 | 22.9 (4.4) | 22.8 (4.5) | 22.9 (4.4) | 0.79 | 32.3 (7.4) | 34.1 (7.8) | 31.4 (7.1) | 0.001 |
| Waist circumference (cm), mean (SD) | 90.4 (12.8) | 89.5 (13.3) | 90.6 (12.7) | 0.66 | 87.3 (11.0) | 86.3 (11.6) | 87.7 (10.8) | 0.23 | 95.0 (21.1) | 96.3 (20.5) | 94.4 (21.4) | 0.47 |
| Hip circumference (cm), mean (SD) | 97.2 (12.7) | 96.0 (14.0) | 97.5 (12.5) | 0.56 | 92.7 (10.1) | 91.9 (10.5) | 93.0 (10.0) | 0.31 | 105.0 (20.5) | 106.8 (21.6) | 104.4 (20.2) | 0.33 |
| Years of schooling, median (IQR) | 7.5 (0, 10) | 8 (0, 11) | 7 (0, 10) | 0.76 | 6 (0, 10) | 7 (0, 10) | 5 (0, 10) | 0.41 | 7 (4, 10) | 7 (5, 9) | 8 (4, 10) | 0.62 |
| Age (years), mean (SD) | 63.2 (11.8) | 57.0 (15.5) | 65.0 (9.9) | <0.001 | 60.0 (11.9) | 50.8 (14.6) | 63.0 (9.1) | <0.001 | 63.7 (10.1) | 61.4 (12.3) | 64.9 (8.6) | 0.002 |
| Gender |  |  |  |  |  |  |  |  |  |  |  |  |
| Female | 100 (56.5%) | 26 (65.0%) | 74 (54.0%) | 0.22 | 262 (47.6%) | 70 (51.1%) | 192 (46.5%) | 0.35 | 239 (64.6%) | 75 (57.3%) | 164 (68.6%) | 0.029 |
| Urban/rural classification |  |  |  |  |  |  |  |  |  |  |  |  |
| *Urban* | 119 (67.2%) | 31 (77.5%) | 88 (64.2%) | 0.12 | 262 (47.6%) | 58 (42.3%) | 204 (49.4%) | 0.15 | 305 (82.7%) | 111 (85.4%) | 194 (81.2%) | 0.31 |
| Ethnicity |  |  |  |  |  |  |  |  |  |  |  |  |
|  | Akan=102 (59.0%) | 17 (47.2%) | 85 (62.0%) | 0.22 | Scheduled tribe/caste=56 (10.3%) | 13 (9.8%) | 43 (10.4%) | 0.60 | African/Black=153 (48.1%) | 39 (49.4%) | 114 (47.7%) | 0.75 |
|  | Ewe=9 (5.2%) | 3 (8.3%) | 6 (4.4%) |  | No caste or tribe=161 (29.5%) | 35 (26.3%) | 126 (30.5%) |  | White=23 (7.2%) | 4 (5.1%) | 19 (7.9%) |  |
|  | Ga-Adangbe=24 (13.9%) | 4 (11.1%) | 20 (14.6%) |  | Other=329 (60.3%) | 85 (63.9%) | 244 (59.1%) |  | Coloured=70 (22.0%) | 16 (20.3%) | 54 (22.6%) |  |
|  | Gruma=15 (8.7%) | 3 (8.3%) | 12 (8.8%) |  |  |  |  |  | Indian/Asian=72 (22.6%) | 20 (25.3%) | 52 (21.8%) |  |
|  | Other=21 (12.1%) | 8 (22.2%) | 13 (9.5%) |  |  |  |  |  |  |  |  |  |
|  | Mole-Dagbon=2 (1.2%) | 1 (2.8%) | 1 (0.7%) |  |  |  |  |  |  |  |  |  |

|  |  | **China** |  |  |  | **Mexico** |  |  |  | **Russia** |  |  |
| --- | --- | --- | --- | --- | --- | --- | --- | --- | --- | --- | --- | --- |
|  | **Full sample** | **Omitted** | **Complete case** | **p*** | **Full sample** | **Omitted** | **Complete case** | **p*** | **Full sample** | **Omitted** | **Complete case** | **p*** |
|  | 869 | 38 | 654 |  | 479 | 164 | 315 |  | 357 | 170 | 187 |  |
| Any leisure-time physical activity |  |  |  |  |  |  |  |  |  |  |  |  |
| *Yes* | 186 (21.5%) | 8 (21.1%) | 142 (21.7%) | 0.92 | 33 (6.9%) | 12 (7.4%) | 21 (6.7%) | 0.76 | 29 (8.2%) | 17 (10.1%) | 12 (6.4%) | 0.20 |
| Any transport physical activity |  |  |  |  |  |  |  |  |  |  |  |  |
| *Yes* | 538 (62.3%) | 25 (65.8%) | 419 (64.1%) | 0.83 | 113 (23.7%) | 32 (19.8%) | 81 (25.7%) | 0.15 | 175 (49.3%) | 77 (45.8%) | 98 (52.4%) | 0.22 |
| Sedentary time (mins), mean (SD) | 262.3 (144.6) | 258.3 (180.6) | 258.0 (141.4) | 0.99 | 166.8 (149.6) | 195.3 (188.2) | 152.0 (122.7) | 0.003 | 330.7 (186.6) | 340.3 (186.9) | 323.1 (186.5) | 0.40 |
| Fruit intake, mean (SD) | 2.0 (1.7) | 2.0 (2.0) | 2.0 (1.7) | 0.93 | 1.6 (1.0) | 1.6 (1.0) | 1.6 (0.9) | 0.84 | 1.4 (1.1) | 1.4 (1.1) | 1.4 (1.0) | 0.97 |
| Vegetable intake, mean (SD) | 6.7 (3.4) | 6.2 (4.1) | 6.6 (3.3) | 0.39 | 1.6 (1.0) | 1.6 (1.0) | 1.6 (1.0) | 0.97 | 1.8 (1.1) | 1.7 (1.2) | 1.8 (1.0) | 0.46 |
| Special diet for diabetes |  |  |  |  |  |  |  |  |  |  |  |  |
| *Yes* | 629 (74.8%) | 24 (64.9%) | 489 (74.8%) | 0.18 | 237 (49.5%) | 88 (53.7%) | 149 (47.3%) | 0.19 | 259 (72.8%) | 118 (69.8%) | 141 (75.4%) | 0.24 |
| Insulin or blood sugar lowering medication |  |  |  |  |  |  |  |  |  |  |  |  |
| *Yes* | 715 (83.3%) | 31 (83.8%) | 541 (82.7%) | 0.87 | 402 (83.9%) | 141 (86.0%) | 261 (82.9%) | 0.38 | 279 (78.2%) | 129 (75.9%) | 150 (80.2%) | 0.32 |
| Body mass index (kg/m2)), mean (SD) | 25.2 (3.6) | 25.9 (3.7) | 24.9 (3.4) | 0.12 | 28.7 (4.7) | 28.9 (4.4) | 28.7 (4.8) | 0.64 | 30.9 (5.4) | 30.3 (5.7) | 31.3 (5.2) | 0.11 |
| Waist circumference (cm), mean (SD) | 88.5 (9.5) | 88.5 (9.6) | 88.2 (9.2) | 0.85 | 99.2 (11.6) | 99.0 (12.5) | 99.3 (11.3) | 0.82 | 100.4 (14.0) | 98.2 (15.2) | 101.6 (13.2) | 0.047 |
| Hip circumference (cm), mean (SD) | 97.8 (7.9) | 97.7 (8.4) | 97.6 (7.7) | 0.97 | 105.4 (10.4) | 104.7 (10.5) | 105.6 (10.4) | 0.47 | 110.6 (12.9) | 110.4 (13.2) | 110.7 (12.8) | 0.83 |
| Years of schooling, median (IQR) | 6 (3, 9) | 9 (6, 11) | 6 (3, 9) | 0.14 | 4 (2, 6) | 5 (2, 8) | 3 (1, 6) | 0.010 | 11 (8, 14) | 11 (8, 14) | 11 (8, 14) | 0.82 |
| Age (years), mean (SD) | 65.3 (9.3) | 52.6 (12.6) | 66.0 (8.8) | <0.001 | 66.4 (10.3) | 63.1 (13.1) | 68.1 (8.0) | <0.001 | 66.5 (10.1) | 66.5 (11.4) | 66.4 (8.9) | 0.94 |
| Gender |  |  |  |  |  |  |  |  |  |  |  |  |
| Female | 496 (57.1%) | 25 (65.8%) | 372 (56.9%) | 0.28 | 332 (69.5%) | 112 (68.7%) | 220 (69.8%) | 0.80 | 278 (77.9%) | 126 (74.1%) | 152 (81.3%) | 0.10 |
| Urban/rural classification |  |  |  |  |  |  |  |  |  |  |  |  |
| *Urban* | 665 (76.5%) | 28 (73.7%) | 511 (78.1%) | 0.52 | 383 (80.0%) | 140 (85.4%) | 243 (77.1%) | 0.033 | 290 (81.2%) | 135 (79.4%) | 155 (82.9%) | 0.40 |
| Ethnicity |  |  |  |  |  |  |  |  |  |  |  |  |
|  | Han=851 (98.2%) | 36 (100.0%) | 640 (97.9%) | 0.38 |  |  |  |  | Russian=282 (82.9%) | 130 (85.0%) | 152 (81.3%) | 0.16 |
|  | Other=16 (1.8%) | 0 (0.0%) | 14 (2.1%) |  |  |  |  |  | Other=23 (6.8%) | 6 (3.9%) | 17 (9.1%) |  |
|  |  |  |  |  |  |  |  |  | Caucasus=35 (10.3%) | 17 (11.1%) | 18 (9.6%) |  |

**Supplementary Table 4.** Country-specific log-binomial regression models of self-reported diabetes diagnosis by years of education^1^.

|  | **Ghana**  **(N = 4152^*^)** | **India**  **(N = 6505^*^)** | **South Africa**  **(N = 3071**^*^**)** | **China**  **(N = 12685^*^)** | **Mexico**  **(N = 1954^*^)** | **Russia**  **(N = 3413**^*^**)** |  |  |  |  |
| --- | --- | --- | --- | --- | --- | --- | --- | --- | --- | --- |
|  | **RR (95% CI)** | **RR (95% CI)** | **RR (95% CI)** | **RR (95% CI)** | **RR (95% CI)** | **RR (95% CI)** |  |  |  |  |
| **Unadjusted** |  |  |  |  |  |  |  |  |  |  |
| Education | 1.09 (1.05, 1.12) | 1.10 (1.08, 1.13) | 1.04 (1.01, 1.07) | 1.05 (1.02, 1.07) | 1.01 (0.98, 1.03) | 0.99 (0.94, 1.04) |  |  |  |  |
| **Adjustment 1** |  |  |  |  |  |  |  |  |  |  |
| Education | 1.09 (1.06, 1.13) | 1.09 (1.07, 1.12) | 1.03 (0.99, 1.06) | 1.02 (0.99, 1.05) | 0.99 (0.96, 1.02) | 1.02 (0.97, 1.07) |  |  |  |  |
| Age | 1.02 (1.01, 1.04) | 1.01 (0.99, 1.03) | 1.02 (1.00, 1.04) | 1.03 (1.02, 1.04) | 0.99 (0.98, 1.01) | 1.02 (1.01, 1.03) |  |  |  |  |
| Gender  *Male*  *Female* | -  1.77 (1.28, 2.45) | -  1.00 (0.80, 1.26) | -  2.01 (1.41, 2.86) | -  1.22 (0.98, 1.51) | -  1.50 (1.09, 2.06) | -  1.39 (0.85, 2.27) |  |  |  |  |
| Urban  *Rural*  *Urban* | -  2.16 (1.43, 3.27) | -  1.79 (1.38, 2.31) | -  2.33 (1.47, 3.70) | -  2.78 (1.95, 3.97) | -  1.73 (1.23, 2.44) | -  1.33 (0.67, 2.64) |  |  |  |  |
|  |  |  |  |  |  |  |  |  |  |  |
| **Adjustment 2**** |  |  |  |  |  |  |  |  |  |  |
| Education | 1.08 (1.04, 1.12) | 1.08 (1.06, 1.11) | 1.02 (0.98, 1.06) | 1.02 (0.99, 1.05) | - | 1.02 (0.97, 1.07) |  |  |  |  |
| Age | 1.02 (1.01, 1.04) | 1.01 (0.99, 1.03) | 1.02 (1.00, 1.03) | 1.03 (1.02, 1.04) |  | 1.02 (1.01, 1.03) |  |  |  |  |
| Gender  *Male*  *Female* | -  1.64 (1.18, 2.30) | -  0.97 (0.78, 1.22) | -  1.99 (1.40, 2.85) | -  1.22 (0.98, 1.51) | - | -  1.39 (0.85, 2.26) |  |  |  |  |
| Ethnicity | *Akan:* -  *Ewe:* 0.71 (0.35, 1.45)  *Ga-Adangbe:* 1.19 (0.72, 1.98)  *Gruma:* 1.40 (0.68, 2.89) *Other:* 0.50 (0.25, 0.98)  *Mole-Dagbon:* 0.38 (0.09, 1.62) | *Scheduled caste/tribe*: -  *No caste/tribe*: 3.99 (2.61, 6.11)  *Other*: 2.15 (1.40, 3.30) | *White:* 1.22 (0.62, 2.38)  *Coloured:* 1.00 (0.65, 1.53)  *Indian/Asian:*2.48 (1.50, 4.09) | *Han:* -  *Other:* 1.12 (0.67, 1.86) | - | *Other:* 1.00 (0.54, 1.86)  *Caucasus:* 1.55 (0.95, 2.52) |  |  |  |  |
| Urban  *Rural*  *Urban* | -  2.12 (1.40, 3.21) | -  1.68 (1.30, 2.17) | -  2.19 (1.37, 3.52) | -  2.78 (1.95, 3.96) | -  - | -  1.32 (0.67, 2.61) |  |  |  |  |
|  |  |  |  |  |  |  |  |  |  |  |

^1^Analysis adjusts for survey weights. ^*^Number of participants in the complete case sample. **Note: No ethnicity data for Mexico so no Adjustment 2.

**Supplementary Table 5.** Sample descriptive characteristics of diabetes self-management factors by country for those with and without diabetes diagnosis.

|  | **Ghana** | | | | **India** | | | **South Africa** | | |
| --- | --- | --- | --- | --- | --- | --- | --- | --- | --- | --- |
|  | | **Diabetes**  **(N = 167)** | **No diabetes**  **(N = 4122)** | **Diabetes**  **(N = 478)** | | **No diabetes**  **(N = 6080)** | **Diabetes**  **(N = 360)** | | **No diabetes**  **(N = 3657)** |  |
| **Fruit per day** | |  |  |  | |  |  | |  |  |
| *Mean (SD)*  *Min – Max*  *Missing, n(%)* | | 2.18 (1.52)  0 – 7  4 (2.4%) | 1.97 (1.48)  0 – 7  134 (3.3%) | 1.16 (0.85)  0 – 3  22 (4.6%) | | 0.79 (0.80)  0 – 3  214 (3.5%) | 1.69 (1.13)  0 – 5  8 (2.2%) | | 1.60 (1.10)  0 – 5  73 (2.0%) |  |
|  | |  |  |  | |  |  | |  |  |
| **Vegetables per day** | |  |  |  | |  |  | |  |  |
| *Mean (SD)*  *Min – Max*  *Missing, n(%)* | | 1.94 (0.86)  0 – 4  6 (3.6%) | 1.98 (0.79)  0 – 4  165 (4.0%) | 2.07 (0.80)  0 – 4  12 (2.5%) | | 1.88 (0.77)  0 – 4  125 (2.06%) | 2.15 (1.20)  0 – 6  6 (1.7%) | | 1.92 (1.11)  0 – 6  76 (2.3%) |  |
|  | |  |  |  | |  |  | |  |  |
| **Fruit and vegetables per day** | |  |  |  | |  |  | |  |  |
| *Mean (SD)*  *Min – Max*  *Missing, n(%)* | | 4.09 (2.09)  0 – 10  8 (4.8%) | 3.93 (1.89)  0 – 11  256 (6.2%) | 3.21 (1.34)  0 – 7  25 (5.2%) | | 2.66 (1.24)  0 – 7  278 (4.6%) | 3.82 (2.11)  0 – 11  11 (3.1%) | | 3.49 (1.93)  0 – 11  113 (3.4%) |  |
|  | |  |  |  | |  |  | |  |  |
| **Body mass index (kg/m^2^)**  *Mean (SD)*  *Min – Max*  *Missing, n(%)* | | 25.82 (5.48)  15.16 – 40.66  10 (6.0%) | 22.84 (4.85)  13.16 – 42.08  136 (3.3%) | 23.00 (4.38)  13.60 – 35.71  16 (3.3%) | | 20.30 (3.97)  11.15 – 37.15  222 (3.7%) | 32.22 (7.43)  15.82 – 59.86  17 (4.7%) | | 29.37 (7.86)  12.87 – 65.00  131 (4.0%) |  |
| **Waist circumference (cm)**  *Mean (SD)*  *Min – Max*  *Missing, n(%)* | | 91.07 (12.52)  52.4 – 120.8  11 (6.6%) | 83.83 (11.56)  48.0 – 122.0  143 (3.5%) | 87.82 (10.93)  53.5 – 117.0  16 (3.4%) | | 81.14 (10.59)  48.00 – 117.0  211 (3.5%) | 94.99 (21.16)  33.00 – 148.00  31 (8.6%) | | 89.80 (20.08)  25.00 – 155.00  173 (5.2%) |  |
|  | |  |  |  | |  |  | |  |  |
| **Hip circumference (cm)**  *Mean (SD)*  *Min – Max*  *Missing, n(%)* | | 98.02 (12.38)  68.00 – 124.6  10 (6.0%) | 91.23 (11.47)  54.00 – 130.4  150 (3.6%) | 92.95 (10.03)  58.00 – 121.00  16 (3.4%) | | 87.98 (9.32)  59.00 – 122.00  211 (3.5%) | 104.65 (20.40)  39.00 – 150.00  39 (10.8%) | | 100.22 (20.81)  32.00 – 170.00  202 (6.1%) |  |
|  | |  |  |  | |  |  | |  |  |
| **Sedentary time (mins/day)**  *Mean (SD)*  *Min – Max*  *Missing, n(%)* | | 257.31 (154.12)  0.00 – 720.00  0 (0.0%) | 219.70 (139.37)  0.00 – 960.00  48 (1.2%) | 192.25 (164.09)  0.00 – 840.00  1 (0.2%) | | 198.52 (153.70)  0.00 – 960.00  13 (0.2%) | 186.30 (137.38)  0.00 – 720.00  4 (1.1%) | | 189.00 (132.38)  0.00 – 960.00  11 (0.3%) |  |
| **Transport physical activity (mins/week)**  *Mean (SD)*  *Min – Max*  *Missing, n(%)* | | 214.91 (274.92)  0.00 – 1260.00  4 (2.4%) | 284.43 (306.98)  0.00 – 1260.00  97 (2.4%) | 264.41 (408.20)  0.00 – 1260.00  14 (2.9%) | | 373.89 (461.41)  0.00 – 1260.00  194 (3.2%) | 99.78 (267.85)  0.00 – 1260.00  14 (3.9%) | | 148.14 (339.15)  0.00 – 1260.00  86 (2.6%) |  |
| **Leisure physical activity (mins/week)**  *Mean (SD)*  *Min – Max*  *Missing, n(%)* | | 51.26 (153.83)  0.00 – 1380.00  4 (2.4%) | 42.99 (137.35)  0.00 – 2100.00  97 (2.4%) | 58.15 (166.65)  0.00 – 1260.00  14 (2.9%) | | 37.11 (149.49)  0.00 – 2160.00  194 (3.2%) | 15.33 (80.62)  0.00 – 900.00  14 (3.9%) | | 22.16 (106.00)  0.00 – 1620.00  86 (2.6%) |  |
|  | |  |  |  | |  |  | |  |  |
|  | |  |  |  | |  |  | |  |  |

|  | **China** | | | | **Mexico** | | | **Russia** | | |
| --- | --- | --- | --- | --- | --- | --- | --- | --- | --- | --- |
|  | | **Diabetes**  **(N = 843)** | **No diabetes**  **(N = 12066)** | **Diabetes**  **(N = 410)** | | **No diabetes**  **(N = 1579)** | **Diabetes**  **(N = 350)** | | **No diabetes**  **(N = 3532)** |  |
| **Fruit per day** | |  |  |  | |  |  | |  |  |
| *Mean (SD)*  *Min – Max*  *Missing, n(%)* | | 1.98 (1.71)  0 – 8  52 (6.2%) | 2.24 (1.84)  0 – 9  1132 (9.4%) | 1.59 (0.97)  0 – 4  12 (2.9%) | | 1.51 (0.93)  0 – 4  74 (4.7%) | 1.40 (1.06)  0 – 5  41 (11.7%) | | 1.46 (1.06)  0 – 5  626 (17.7%) |  |
|  | |  |  |  | |  |  | |  |  |
| **Vegetables per day** | |  |  |  | |  |  | |  |  |
| *Mean (SD)*  *Min – Max*  *Missing, n(%)* | | 6.75 (3.39)  0 – 18  41 (4.9%) | 6.67 (3.45)  0 – 19  705 (5.8%) | 1.63 (0.96)  0 – 4  14 (3.4%) | | 1.59 (0.92)  0 – 4  83 (5.3%) | 1.76 (1.11)  0 – 6  38 (10.9%) | | 1.81 (1.06)  0 – 6  659 (18.7%) |  |
|  | |  |  |  | |  |  | |  |  |
| **Fruit and vegetables per day** | |  |  |  | |  |  | |  |  |
| *Mean (SD)*  *Min – Max*  *Missing, n(%)* | | 8.60 (3.87)  0 – 24  83 (9.8%) | 8.82 (4.06)  0 – 26  1428 (11.8%) | 3.20 (1.55)  0 – 8  24 (5.9%) | | 3.06 (1.52)  0 – 8  125 (7.9%) | 3.14 (1.84)  0 – 10  52 (14.9%) | | 3.24 (1.79)  0 – 11  772 (21.9%) |  |
|  | |  |  |  | |  |  | |  |  |
| **Body mass index (kg/m^2^)**  *Mean (SD)*  *Min – Max*  *Missing, n(%)* | | 25.13 (3.55)  15.56 – 38.06  52 (6.2%) | 23.65 (3.41)  13.30 – 38.44  622 (5.2%) | 28.69 (4.79)  18.00 – 43.32  58 (14.1%) | | 27.87 (4.83)  15.05 – 43.66  171 (10.8%) | 31.05 (5.28)  19.21 – 45.34  40 (11.4%) | | 28.33 (4.93)  16.04 – 46.68  393 (11.1%) |  |
| **Waist circumference (cm)**  *Mean (SD)*  *Min – Max*  *Missing, n(%)* | | 88.51 (9.53)  63.00 – 115.40  52 (6.2%) | 83.89 (9.72)  54.00 – 115.10  541 (4.5%) | 99.72 (11.71)  69.00 – 134.20  53 (12.9%) | | 96.31 (11.98)  61.20 – 135.20  150 (9.5%) | 100.69 (13.70)  60.00 – 140.00  66 (18.9%) | | 93.75 (14.08)  50.00 – 140.10  740 (21.0%) |  |
|  | |  |  |  | |  |  | |  |  |
| **Hip circumference (cm)**  *Mean (SD)*  *Min – Max*  *Missing, n(%)* | | 97.82 (7.92)  73.00 – 120.00  51 (6.0%) | 94.70 (7.76)  70.00 – 120.00  601 (5.0%) | 105.64 (10.51)  85.7 – 137.4  55 (13.4%) | | 103.27 (10.58)  76.8 – 138.5  161 (10.2%) | 110.59 (12.87)  70.00 – 146.10  74 (21.1%) | | 104.25 (14.24)  58.00 – 150.30  766 (21.7%) |  |
|  | |  |  |  | |  |  | |  |  |
| **Sedentary time (mins/day)**  *Mean (SD)*  *Min – Max*  *Missing, n(%)* | | 262.40 (143.57)  0.00 – 960.00  34 (4.0%) | 222.65 (133.92)  0.00 – 960.00  290 (2.4%) | 171.69 (152.00)  0.00 – 840.00  1 (0.2%) | | 149.07 (128.60)  0.00 – 780.00  11 (0.7%) | 333.18 (187.04)  30.00 – 960.00  23 (6.6%) | | 288.80 (176.30)  0.00 – 960.00  207 (5.9%) |  |
| **Transport physical activity (mins/week)**  *Mean (SD)*  *Min – Max*  *Missing, n(%)* | | 196.36 (265.17)  0.00 – 1260.00  5 (0.6%) | 202.88 (276.35)  0.00 – 1260.00  108 (0.9%) | 159.96 (357.26)  0.00 – 1260.00  1 (0.2%) | | 187.11 (393.94)  0.00 – 1260.00  13 (0.8%) | 363.97 (454.34)  0.00 – 1260.00  2 (0.6%) | | 450.45 (470.91)  0.00 – 1260.00  37 (1.0%) |  |
| **Leisure physical activity (mins/week)**  *Mean (SD)*  *Min – Max*  *Missing, n(%)* | | 88.04 (216.57)  0.00 – 1680.00  5 (0.6%) | 71.20 (222.70)  0.00 – 2520.00  108 (0.9%) | 15.99 (91.61)  0.00 – 1260.00  1 (0.2%) | | 20.84 (104.28)  0.00 – 1260.00  13 (0.8%) | 17.76 (81.46)  0.00 – 840.00  2 (0.6%) | | 27.86 (124.75)  0.00 – 2100.00  37 (1.0%) |  |
|  | |  |  |  | |  |  | |  |  |
|  | |  |  |  | |  |  | |  |  |

^*^Note: Due to extreme values for some variables, outliers (values ±3×SD from the mean) were recoded to missing for fruit, vegetables, BMI, waist circumference, hip circumference. Physical activity and sedentary variables were cleaned according to GPAQ cleaning guidelines.
